# Diagnosis, treatment and postsurgical complications in a dog with epileptic seizures and a naso-ethmoidal meningoencephalocele

**DOI:** 10.1186/s13028-021-00591-1

**Published:** 2021-07-08

**Authors:** Abtin Mojarradi, Sofie Van Meervenne, Alejandro Suarez-Bonnet, Steven De Decker

**Affiliations:** 1 The IVC Evidensia Referral Hospital in Helsingborg, Bergavagen 3, 25466 Helsingborg, Sweden; 2 Anicura Kalmar Small Animal Clinic, Gasverksgatan 17, 39245 Kalmar, Sweden; 3 Department of Pathobiology and Population Sciences, Royal Veterinary College, University of London, Hawkshead lane, North Mymms, Hatfield, AL9 7TA UK; 4 Department of Clinical Science and Services, Royal Veterinary College, University of London, Hawkshead lane, North Mymms, Hatfield, AL9 7TA UK

**Keywords:** Apergillosis, Pneumocephalus, Pneumorrhachis, Transfrontal craniotomy

## Abstract

**Background:**

Naso-ethmoidal meningoencephalocele is usually a congenital anomaly consisting of a protrusion of cerebral tissue and meninges into the ethmoidal labyrinth. The condition is a rare cause of structural epilepsy in dogs. We report the clinical presentation, surgical intervention, postoperative complications and outcome in a dog with drug resistant epilepsy secondary to a meningoencephalocele.

**Case presentation:**

A 3.3-year-old male neutered Tamaskan Dog was referred for assessment of epileptic seizures secondary to a previously diagnosed left-sided naso-ethmoidal meningoencephalocele. The dog was drug resistant to medical management with phenobarbital, potassium bromide and levetiracetam. Surgical intervention was performed by a transfrontal craniotomy with resection of the meningoencephalocele and closure of the dural defect. Twenty-four hours after surgery the dog demonstrated progressive cervical hyperaesthesia caused by tension pneumocephalus and pneumorrhachis. Replacement of the fascial graft resulted in immediate resolution of the dog’s neurological signs. Within 5 months after surgery the dog progressively developed sneezing and haemorrhagic nasal discharge, caused by sinonasal aspergillosis. Systemic medical management with oral itraconazole (7 mg/kg orally q12h) was well-tolerated and resulted in resolution of the clinical signs. The itraconazole was tapered with no relapsing upper airway signs. The dog’s frequency of epileptic seizures was not affected by surgical resection of the meningoencephalocele. No treatment adjustments of the anti-epileptic medication have been necessary during the follow-up period of 15 months.

**Conclusions:**

Surgical resection of the meningoencephalocele did not affect the seizure frequency of the dog. Further research on prognostic factors associated with surgical treatment of meningoencephaloceles in dogs is necessary. Careful monitoring for postsurgical complications allows prompt initiation of appropriate treatment.

## Background

A meningoencephalocele (MEC) is defined as a protrusion of cerebral tissue and meninges through a defect of the cranial bones [1–6]. It is usually a congenital anomaly that is hypothesized to be caused by a neural tube defect where the neuroectoderm and surface ectoderm fail to separate [4, 6–8]. The pathogenesis for congenital MECs in humans is multifactorial, including genetic and environmental risk factors [8]. In people, MECs are classified according to the localization of the skull defect, with intranasal protrusion of cerebral tissue, into the ethmoidal labyrinth, being named a naso-ethmoidal MEC [5]. The pathology is considered rare in veterinary medicine but has been described in a number of dogs with the most common clinical signs being epileptic seizures and behavioural abnormalities [3, 9–11]. Successful surgical treatment of a naso-ethmoidal MEC in a puppy with epileptic seizures, resulting in seizure-freedom, has been reported [3]. We report the clinical signs, imaging, surgical treatment, postoperative complications and outcome of a naso-ethmoidal MEC in a dog with drug resistant epilepsy.

## Case presentation

A 3.3-year-old male intact Tamaskan Dog, weighing 29 kg, was referred to the IVC Evidensia Referral Hospital in Helsingborg, Sweden for further assessment of epileptic seizures. The dog experienced its first epileptic seizure at the age of 4 months. Haematology, serum biochemistry profile, including ammonia and bile acids, and urinary analysis obtained at that time were all within normal limits. There was a history of littermates of the dog experiencing epileptic seizures, without any further information regarding diagnostic tests or response to treatment. Despite treatment with phenobarbital the dog continued to demonstrate generalised epileptic seizures every 10–14 days. At the age of 7 months the dog underwent magnetic resonance imaging (MRI; 0.25 T Vet-MR Grande, Esaote, Genoa, Italy) of the head, which revealed a left-sided naso-ethmoidal MEC. Despite a therapeutic serum concentration of 36 mg/L (therapeutic range 15–40 mg/L [12]) of phenobarbital, the dog continued to demonstrate epileptic seizures with the same frequency. Adding treatment with potassium bromide (serum concentration 1700 mg/L: therapeutic range 1000–2000 mg/L [12]) and levetiracetam (17 mg/kg, orally q8h) did not result in improved epileptic seizure control. The dog was considered drug resistant and was therefore referred for surgical intervention.

**Fig. 1:**
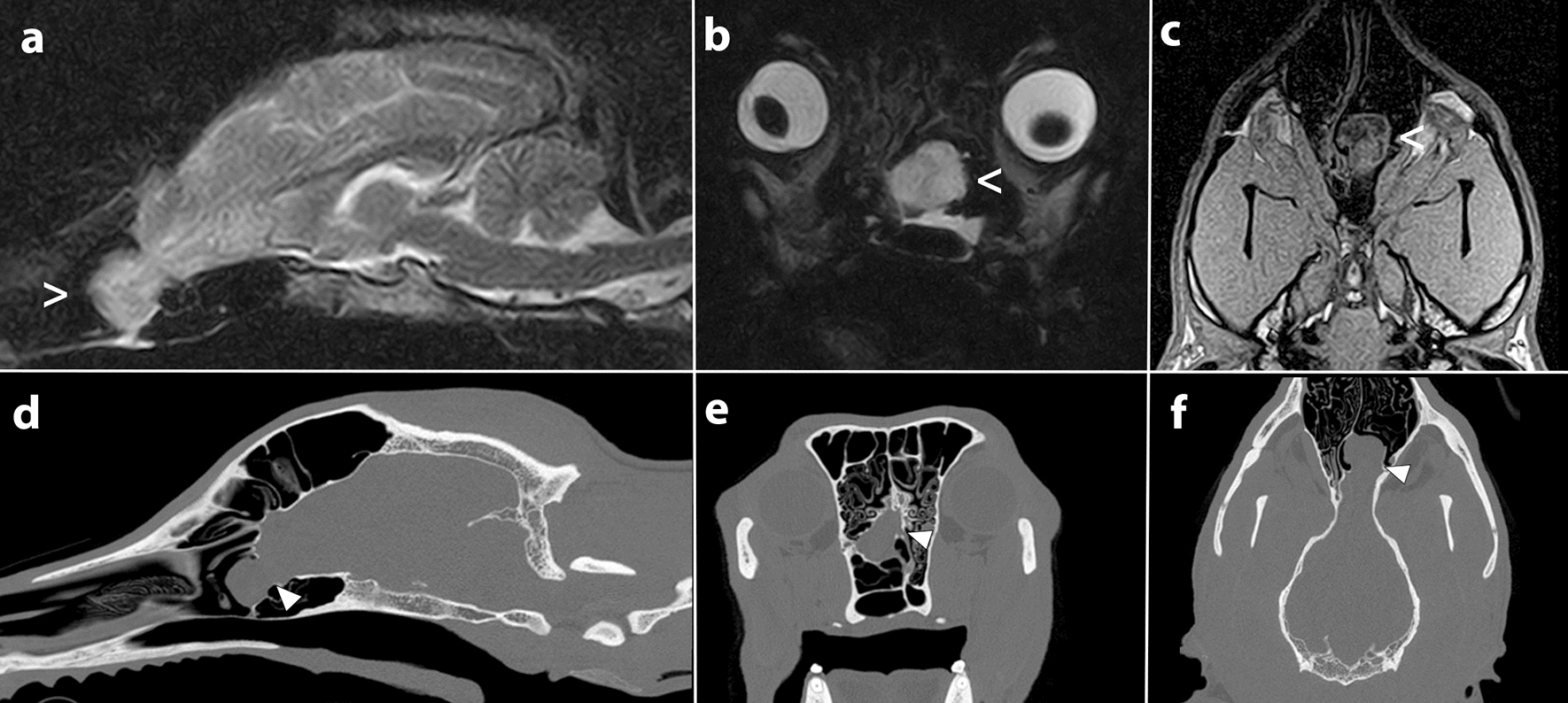
Magnetic resonance imaging (MRI) and computed tomography (CT) of a dog’s head. Sagittal T2-weighted (**a**), transverse T2-weighted (**b**) and dorsal T1-weighted (**c**) sequences; bone reconstruction in sagittal (**d**), transverse (**e**) and dorsal (**f**) orientation. MRI and CT show a left sided naso-ethmoidal meningoencephalocele (empty arrowhead) protruding through a defect of the cribriform plate (filled arrowhead)

On presentation, the general physical and neurological examination were within normal limits. The dog was currently on treatment with phenobarbital (Fenemal, 5.2 mg/kg orally q12h; Meda A/S, Solna, Sweden) and potassium bromide (Libromide, 11 mg/kg orally q12h; Dechra Veterinary Products A/S, Uldum, Denmark) yielding serum concentrations of 36 mg/L and 1180 mg/L, respectively. For surgical planning, the dog had a computed tomography (CT) and a new MRI performed 1 month before the surgery. The dog was premedicated with 0.1 mg/kg butorphanol (Butomidor vet; Salfarm Scandinavica A/S, Helsingborg, Sweden) in combination with 7 µg/kg medetomidine (Sedator vet; Dechra Veterinary Products A/S) IV. General anaesthesia was induced with 1.4 mg/kg propofol (Propovet Multidose; Zoetis Inc., NY, USA) IV and maintained with isoflurane vaporized in oxygen. An MRI (0.25 T Vet-MR Grande, Esaote, Genoa, Italy) study including sagittal T2-weighted [repetition time (TR: ms)/echo time (ms) (TE), 3770/120], transverse T2-weighted (TR/TE, 7270/120), transverse T1-weighted (TR/TE, 550/18), dorsal T1-weighted (TR/TE, 22/9), dorsal FLAIR (TR/TE, 6090/120) and dorsal HYCE (TR/TE, 10/5) sequences. T1-weighted images were acquired before and after IV administration of gadolinium-based contrast medium (Dotarem; Gothia Medical A/S, Billdal, Sweden). Immediately after MRI, CT (Philips Brilliance Big Bore, 16 slice, helical mode, 1-mm slice thickness, 0.5 mm overlap between slices, 120 kVp, 400 mA, bone and soft tissue reconstruction algorithms) was performed of the dog’s head. The MRI and CT confirmed the diagnosis of naso-ethmoidal MEC with the rostral part of the left olfactory bulb protruding into the nasal cavity through a defect in the cribriform plate (Fig. 1).

**Fig. 2:**
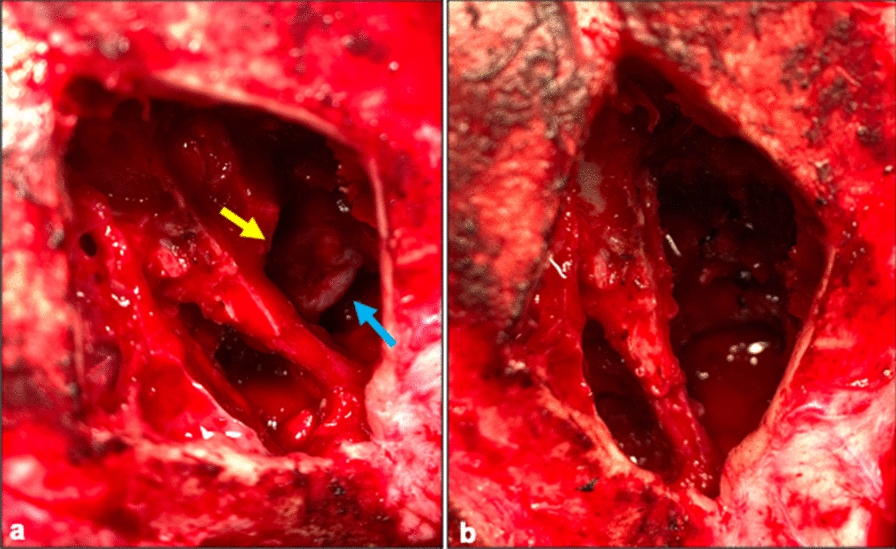
Intra-operative pictures before (**a**) and after (**b**) surgical resection of a naso-ethmoidal meningoencephalocele. Brain tissue is protruding into the nasal cavity (blue arrow) through a defect in the cribriform plate (yellow arrow; **a**). The protruding tissue is resected by use of bipolar cautery (**b**)

For surgery, the dog was premedicated with 4 µg/kg medetomidine **(**Sedator vet; Dechra Veterinary Products A/S) in combination with 0.2 mg/kg methadone (Semfortan vet; Dechra Veterinary Products A/S) IV, induced with 2.2 mg/kg propofol (Propovet Multidose; Zoetis Inc.) IV and general anaesthesia was maintained with isoflurane vaporized in oxygen. To access the MEC, a modified bilateral transfrontal craniotomy was performed [13] using an oscillating bone saw to make a diamond-shaped bone flap overlying the frontal sinus. Sinus septa and ethmoturbinates were removed with rongeurs and the MEC was resected by bipolar cautery (Fig. 2). An autologous fascial graft was harvested from the right temporal muscle and used together with a topical tissue adhesive (GLUture; Zoetis Inc.) to seal the calvarium. The original bone flap was replaced and sutured through pre-drilled burr holes with monofilament nonabsorbable suture (Ethilon 3–0; Ethicon Inc., NJ, USA). The edges of the replaced bone flap and sutured burr holes were covered by a mixture of beeswax and vaseline (Bone Wax; B. Braun Medical A/S, Melsungen, Germany). The surgical wound was routinely closed (Monocryl 3–0; Ethicon Inc.) and protected with an adhesive cover (Primapore; Smith & Nephew A/S, London, UK). Pain management during surgery was maintained with a constant rate infusion (CRI) of 10 µg/kg/h fentanyl (Fentadon vet; Dechra Veterinary Products A/S).

**Fig. 3:**
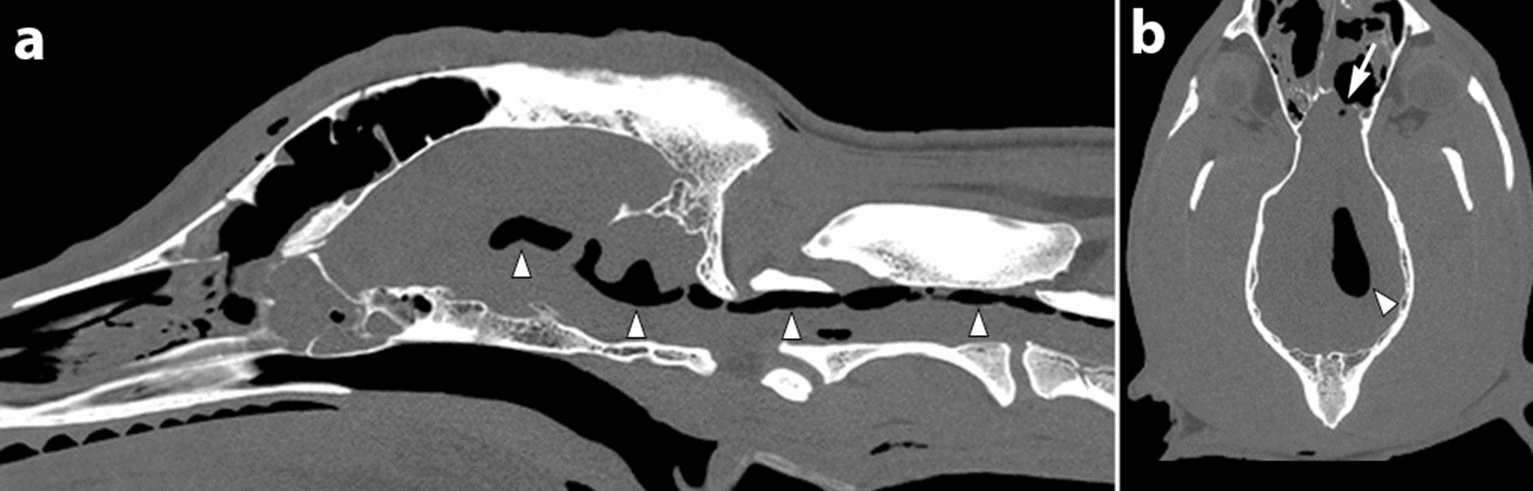
Computed tomography (CT) of a dog’s head and craniocervical vertebral column. Bone reconstruction in mid-sagittal (**a**) and dorsal (**b**) orientation. Gas accumulation is seen in the left lateral ventricle (filled arrowhead; **b**), the third ventricle, the fourth ventricle and the cervical dorsal subarachnoid space (filled arrowhead; **a**). Resection of the meningoencephalocele has been successfully accomplished (arrow; **b**)

Postoperatively, the dog received dexamethasone (Dexadreson vet, 0.1 mg/kg/IV q24h; Intervet A/S, Stockholm, Sweden), methadone (Semfortan vet, 0.2 mg/kg IV q4h; Dechra Veterinary Products A/S), maropitant (Prevomax, 1 mg/kg IV q24h; Dechra Veterinary Products A/S), paracetamol (Paracetamol 10 mg/kg IV q12h; B. Braun Medical A/S) and phenobarbital (Fenemal, 5.1 mg/kg IV q12h; Meda AB). The dog recovered uneventfully and his neurological examination was unremarkable 12 h after surgery. Twenty-four hours after surgery the dog started to demonstrate progressive cervical hyperaesthesia unresponsive to an IV CRI of fentanyl (Fentadon vet, 10 mcg/kg/h CRI; Dechra Veterinary Products A/S). A tension pneumocephalus and pneumorrhachis was suspected and confirmed by CT 36 h after surgery (Fig. 3).

For revision surgery, the dog was premedicated with 0.2 mg/kg/IV diazepam (Stesolid novum; Teva A/S, Petah Tikva, Israel), induced with 3.4 mg/kg/IV propofol (Propovet Multidose; Zoetis Inc.) and maintained with isoflurane vaporized in oxygen. The dog continued on its previous CRI 10 mcg/kg/h of fentanyl (Fentadon vet; Dechra Veterinary Products A/S) during surgery. Close inspection of the fascial graft revealed a possible defect in the ventral part of the graft. The graft was removed and replaced with a new fascial graft, harvested from the left temporalis muscle, attached with topical tissue adhesive (GLUture; Zoetis Inc.). Replacement of the bone flap, and closure of the surgical wound, was performed as previously described. The dog recovered uneventfully, was neurologically normal without any signs of discomfort 4 h after revision surgery, and was discharged from hospitalisation 2 days later. Treatment at home consisted of a tapering anti-inflammatory dose of prednisone (Prednisolon, 1 mg/kg orally q24h, 2 weeks; Pfizer A/S, NY, USA) and paracetamol (Alvedon, 15 mg/kg orally q12h, 1 week; GSK Consumer Healthcare A/S, Brentford, UK). The dog continued on its previous antiepileptic treatment with phenobarbital and potassium bromide.

**Fig. 4:**
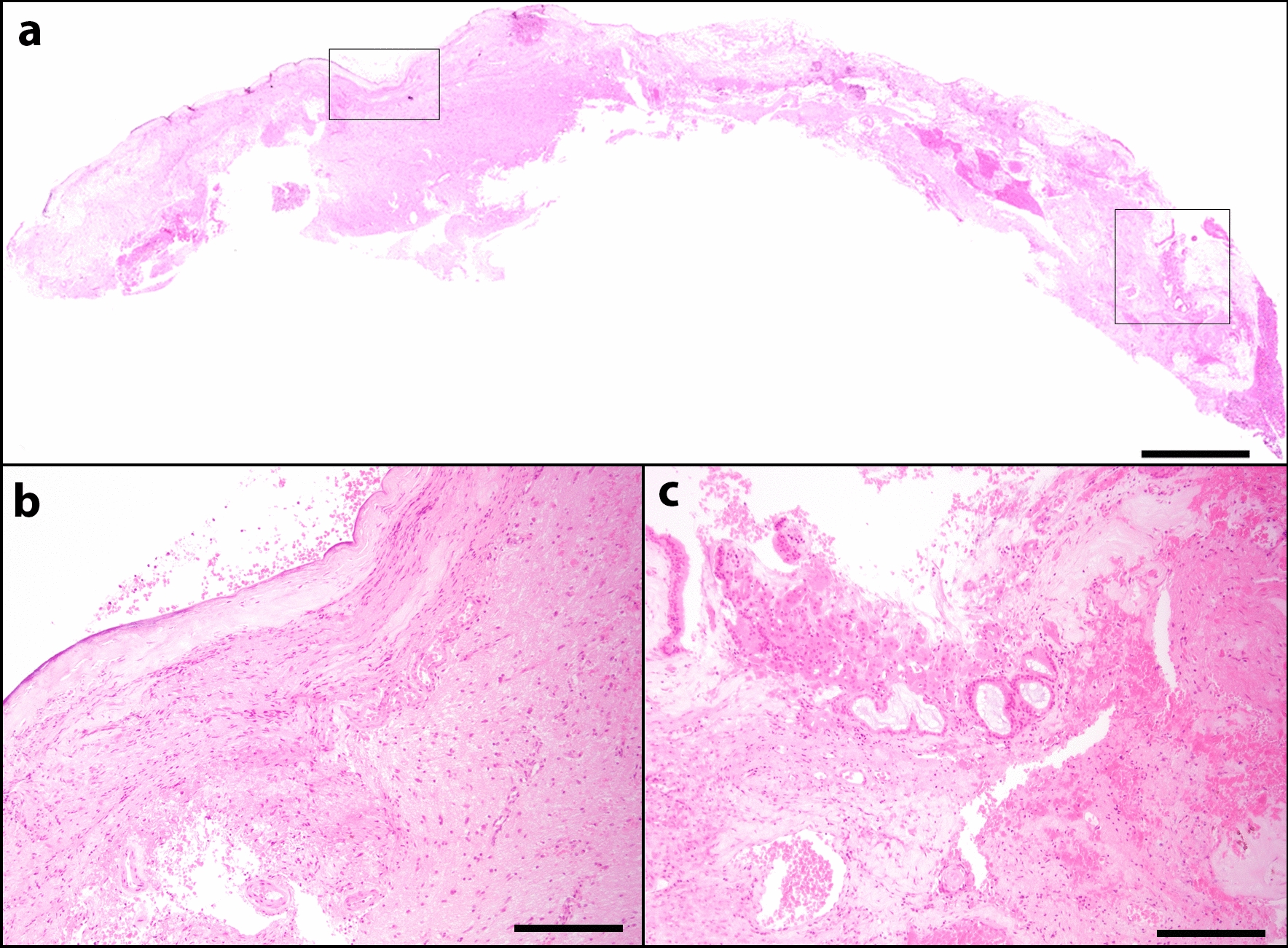
Microscopic appearance of a resected unilateral naso-ethmoidal meningoencephalocele (**a**, scale bar  =  1000 µm) with left (**b**, scale bar  =  50 µm) and right (**c**, scale bar  =  50 µm) box magnified. Subgrossly the section is mildly fragmented and exhibits multifocal areas of haemorrhage. The neuropil is caudally lined by a thick layer of fibrous and mildly mineralized connective tissue (**b**). The neuropil is lined by respiratory epithelium that is multifocally loss and replaced by haemorrhage and necrotic debris (**c**)

Histopathology (Fig. 4) of the resected fragment revealed fragmented and haemorrhagic neuropil lined rostrally by respiratory, tall columnar, pseudostratified, ciliated epithelium. The underlying neuropil was multifocally rarefied and blood vessels where occasionally expanded by neutrophils and lymphocytes. Occasional oligodendrocytes and astrocytes (gliosis) were scattered within the olfactory bulb. More caudally the neuropil was lined by a thick layer of regular dense and superficially mineralized fibrous connective tissue, consistent with a fibrous dura.

**Fig. 5:**
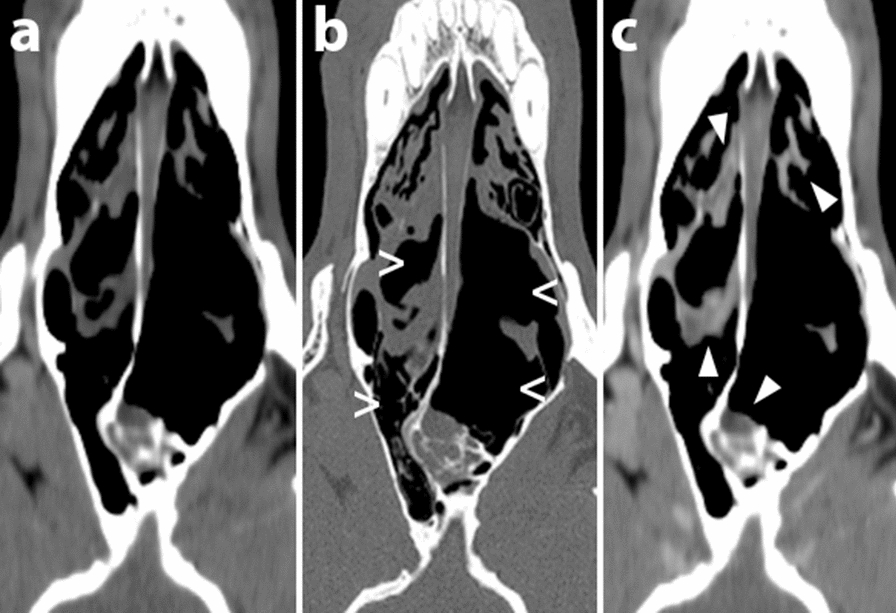
Computed tomography of a dog’s nasal cavity. Soft tissue reconstruction pre (**a**) and post-contrast (**c**) and bone reconstruction (**b**) in dorsal orientation show marked bilateral soft tissue inflammation (filled arrowhead; **a**, **c**) and severe bilateral destruction of the nasal turbinates (empty arrowhead; **b**) indicative of sinonasal aspergillosis

General physical and neurological examinations 14 days after surgery did not reveal any abnormalities. The dog had experienced one epileptic seizure since the surgery. The dog developed mucoid nasal discharge within 2 months, that progressed to haemorrhagic mucopurulent nasal discharge within 5 months, after surgery. A CT, pre and post-contrast (Optiray, 600 mg/kg IV; Gothia Medical A/S), of the dog’s head (Fig. 5), showed bilateral destructive rhinitis with suspected fungal infection of the bone flap. A revision surgery was performed where the bone flap was removed and sent for histopathology. The nasal cavity was flushed under pressure with 500 ml of warm saline. Samples for cytology, histopathology and mycotic culture were obtained, and serology for aspergillosis was analyzed.

Serology for aspergillosis was negative. Cytology was indicative of aspergillosis which was confirmed by mycotic culture (*Aspergillus fumigatus*) and histopathology. Treatment with itraconazole (Itrakonazol Actavis, 7 mg/kg orally q12h; Teva A/S) was initiated, which resulted in resolution of clinical signs. Tapering of the treatment, after 3 months, resulted in relapsing upper airway signs. The itraconazole was reinstituted and another CT was performed after a total of 7 months on treatment with itraconazole. On CT, the previous inflammatory findings of the soft tissue in the nasal cavity had resolved. During rhinoscopy, a suspected fungal granuloma was removed and sent for histopathology, in combination with cytology and mycotic culture, which all came back negative for aspergillosis. Treatment with itraconazole was tapered and stopped 3 months later, without relapse of upper airway signs. At last re-examination at 15 months after surgery, the dog continued having a frequency of one epileptic seizure every 10–14 days. No adjustments of the anti-epileptic medical treatment have been made since the surgery of the MEC.

## Discussion and conclusions

The surgical treatment of a naso-ethmoidal MEC, including the development and management of tension pneumocephalus, pneumorrhachis and sinonasal aspergillosis, in a dog is reported. Even though a traumatic MEC cannot be excluded [4], the dog’s young age at diagnosis makes a congenital aetiology more likely. The anomaly is considered rare in dogs with only a limited number of cases being reported [3, 9–11]. Although neurological signs caused by MECs in humans are reported rare, the most common neurological sign reported is epileptic seizures [1, 2], which corresponds to reported findings in dogs [3, 9–11]. Encephaloceles in people are considered epileptic foci which may result in refractory epilepsy [1, 2, 14] and surgical treatment with resection of the MEC and reconstruction of the bone defect is considered the treatment of choice [1, 6, 15]. This surgical approach may result in elimination of epileptic seizures in humans [1, 2, 14, 16]. Dogs with MECs may respond to medical management with anti-epileptic drugs [9] but drug resistant disease, as in the reported case, is a possible indication for surgical management [3, 9]. Unfortunately, the dog’s frequency of epileptic seizures did not decrease during the postoperative follow-up period of 15 months. Although the dog’s frequency and severity of epileptic seizures did not deteriorate further, the surgery was considered unsuccessful regarding its primary objective. In the previously reported case of a successful surgical management of a naso-ethmoidal MEC in a dog that yielded seizure-freedom, surgery was performed when the dog was 5 months old and one month after onset of epileptic seizures [3]. This is in contrast to the case reported here in which surgery was performed 36 months after the onset of epileptic seizures. It is possible that the dog’s history of drug resistant epileptic seizures before surgery had resulted in development of additional epileptic foci attributed to mirror focus and kindling phenomenon [17, 18]. It can therefore not be excluded that surgical treatment soon after the time of diagnosis would have resulted in a different outcome. Furthermore, the previously described case did not have histopathological abnormalities detected on the resected MEC [3], while the dog in this case had histopathological findings of inflammation, haemorrhage and neuropil degeneration. There is a possibility that not all abnormal brain tissue was removed at surgery. A more aggressive approach would possibly have yielded a different outcome, but there is some controversy in human medicine considering the extent of surgical resection with no consensus being reached [1, 16]. A secondary aim of the surgery was to close the dural defect, thereby decreasing the risk for ascending meningitis [15, 19]. After the revision surgery, where the fascial graft was replaced, the dog recovered uneventfully from its pneumocephalus and pneumorrhachis, indicating a complete closure of the defect and success of the secondary aim.

In the immediate postoperative period, the dog developed progressive cervical hyperaesthesia. The occurrence of progressive cervical hyperaesthesia in the early post-operative period after a craniotomy should be considered suggestive for development of tension pneumocephalus and pneumorrhachis [20]. Pneumocephalus and pneumorrhachis are defined as gas accumulation within the cranial cavity and the vertebral canal, respectively [21, 22]. Pneumocephalus in itself is asymptomatic, occurs in up to 100% of humans after craniotomy, and resolves without surgical intervention [23]. When clinical signs occur associated with this gas accumulation, the condition is referred to as tension pneumocephalus [23, 24]. This is however an uncommon condition in human and veterinary medicine [20, 21, 24–26]. The use of CT for detection of pneumocephalus and pneumorrhachis is more sensitive than MRI and was used in the presented case [27, 28]. The pathogenesis of tension pneumocephalus is not clear and several theories have been discussed [29]. Positive (ball-valve) and negative pressure (inverted bottle and hydrodynamic) theories have been proposed to explain the phenomenon [21, 30]. All theories are dependent on an extra- and intracranial communication through a bony defect, a tear of the dura and a fistula in the brain parenchyma [21]. In the reported case, a defect in the fascial graft was the likely extra- and intracranial communication to facilitate gas accumulation in the brain and subarachnoid space. A tension pneumocephalus is considered an emergency due to progressive compression of neural tissue ultimately leading to cerebral and/or brainstem herniation [26]. There were no findings on CT that would indicate brain herniation, but surgical replacement of the fascial graft, thereby restoring the integrity of the cranial vault, resulted in immediate resolution of the dog’s neurological signs. This development is in agreement with the literature, where surgical closure of the dural defect typically carries a good prognosis with gradual resolution of neurological signs [20, 25, 26, 29, 31–33].

The dog developed clinical signs of rhinitis within months after surgery, which progressed to haemorrhagic mucopurulent nasal discharge. Nasal discharge, sneezing and epistaxis are well known clinical signs of sinonasal aspergillosis in dogs [34]. With the use of imaging, cytology, histology and mycotic culture a diagnosis of sinonasal aspergillosis caused by A. *fumigatus* was established [34–38]. A. *fumigatus* is an opportunistic pathogen which may cause sinonasal aspergillosis secondary to previous nasal trauma and the presence of foreign bodies [39]. We hypothesize that the pathogen colonized and invaded the nasal mucosa secondary to the surgical trauma and it can be considered if the topical tissue adhesive functioned as a foreign body, further enabling colonization. It is also possible that the bone flap functioned as a locus minoris resistentiae, which would explain the severe fungal infection of the bone flap that was demonstrated during the revision surgery. When removing the bone flap, and flushing the nasal cavity with saline, the dog’s clinical signs immediately improved. Topical local treatment was not administered since an intracranial communication, risking potentially severe complications, could not be completely excluded [35]. The use of oral itraconazole as systemic treatment for sinonasal aspergillosis has been described with a reported efficiency in 60–70% of infected dogs, with side-effects including hepatotoxicity, anorexia and gastrointestinal signs [35, 40]. The treatment was well-tolerated by the dog and considered effective with resolution of the clinical signs and follow-up diagnostic imaging not being able to demonstrate residual infection after 7 months of treatment. Tapering of the treatment over 3 months was well-tolerated and the dog has not demonstrated any signs of relapse after stopping the treatment.

As final conclusions for this case, the need for further research regarding the use of surgical treatment for MECs in dogs is warranted. It can be argued that surgical treatment should preferably be performed at a young age. In the immediate postoperative period, the clinician should closely monitor the dog for progressive neurological signs, indicating the possible presence of tension pneumocephalus and pneumorrhachis. Surgical treatment of pneumocephalus and pneumorrhacis may carry an excellent prognosis, depending on the underlying cause, and may yield an immediate treatment response. Furthermore, although this dog developed sinonasal aspergillosis after transfrontal craniotomy, the relationship between proliferation of this opportunistic pathogen and surgery affecting the frontal sinus remains unclear.

## Data Availability

The datasets used and/or analysed during the current study are available from the corresponding author on reasonable request.

## Abbreviations

CT: Computed tomography
CRI: Constant rate infusion
IM: Intramuscularly
IV: Intravenously
MEC: Meningoencephalocele
MRI: Magnetic resonance imaging
q4h: Six times a day
q6h: Four times a day
q12h: Two times a day
q24h: One time a day
TE: Time of echo
TR: Time of repetition
